# Supplementary material for: Transcriptome Analysis Provides Insights into the Mechanism of Astaxanthin Enrichment in a Mutant of the Ridgetail White Prawn Exopalaemon carinicauda
Source: Genes (Basel). 2021 Apr 21;12(5):618. doi: 10.3390/genes12050618 (PMC8143343; doi:10.3390/genes12050618)
Supplement: Supplementary file 1 [file genes-12-00618-s001.zip › Figure S1-2.pdf]

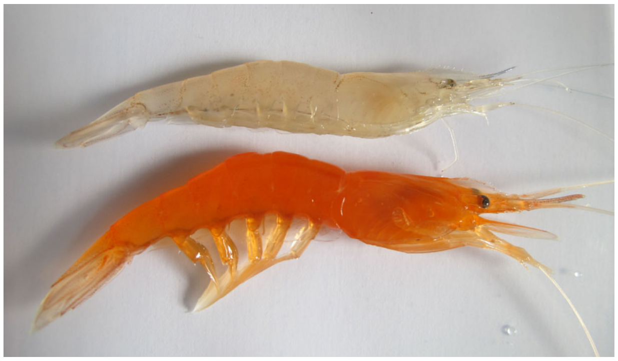

**Figure S1**. Figure of the wild-type prawn (up) and the orange-red variant prawn (down).

```
Unigene0036561     -------MLPFLIAACALFSTTALGHIDVGVCPDIVDKYDFDPVPYLGRWYEAQRFHIVY
Unigene0044620     MFEYLYLLSAALVTVSHGMIISGEPKVDVGQCPNITNKYDFEAAPYLGVWYELERFPMSF
Unigene0028810     ---------------MNLNQMTSGQVFFRGPCPDIPPLKDFQPKRFLGRWYEIERFFVSY
                                        :.   .  * **:*    **:.  :** *** :** : :

Unigene0036561     E--IGQDCVNTIYDDLGDGYIGVHNMGRSASGNLTDISG--KAHLIAPG--VLSVEFLGY
Unigene0044620     E--AGQDCVTATYSVLDDVYIKVYNRARLLKGPIVDITG--RAHVIAPG--VLLVEFFTM
Unigene0028810     EDLAGSCWVENYLFEKGRGHFTRLDWKDHLSGRILSIENGITFGKKEPGRLRYVVQRPSI
                   *   *.  *       .  ::   :     .* : .* .        **     *:

Unigene0036561     PAG--DYHVLGTDYENFSCVYDCEQD--GPLRTQHAWLLTRAKIPDEATFEIANEVFARN
Unigene0044620     ENSGAEYHVLDTDYEKFACVYNCVQT--GPVRAQYAWLLSRTTDMDDETYNHAMGVFTDN
Unigene0028810     PILQGEYQILATDYYSYALAWQCMDFPLSLGHTEILWFLSKEQFPSYETEQTAKRLAAKF
                        :*::* *** .:: .::* :   .  :::  *:*::    .  * : *  : :

Unigene0036561     GIDVSLFHRTHQGDDCPYVV
Unigene0044620     GIDISLFQTTYQASDCPYLE
Unigene0028810     GLHVKSLQKQDR-THCPL--
                   *:.:. ::   :  .**
```

**Figure S2.** Alignment of Apolipoprotein D amino acid sequences. Unigene0036561 and Unigene0044620, which were all with lower expression level in the orange-red variant, shared 65.63% sequence identity. Unigene0028810 which was with lower expression level in the orange-red variant, shared only 40.61% and 37.31% sequence identity with Unigene0036561 and Unigene0044620, respectively.
