# Supplementary material for: Transcriptome Analysis Provides Insights into the Mechanism of Astaxanthin Enrichment in a Mutant of the Ridgetail White Prawn Exopalaemon carinicauda
Source: Genes (Basel). 2021 Apr 21;12(5):618. doi: 10.3390/genes12050618 (PMC8143343; doi:10.3390/genes12050618)
Supplement: Supplementary file 1 [file genes-12-00618-s001.zip › Table S1.pdf]

Table S1. Information of DEGs with higher expression level in EcR prawns.

GeneID	Description	Species
Unigene0075750	Proteasome subunit alpha type-4	*Trachymyrmex septentrionalis*
Unigene0025033	Proline-rich protein	*Thecamonas trahens ATCC 50062*
Unigene0075630	Hypothetical protein PC101070.00.0, partial	*Plasmodium chabaudi chabaudi*
Unigene0050620	Hypothetical protein BRAFLDRAFT_92590	*Branchiostoma floridae*
Unigene0076150	SJCHGC01393 protein	*Schistosoma japonicum*
Unigene0027566	Uncharacterized protein LOC102810112	*Saccoglossus kowalevskii*
Unigene0055895	Decapping and exoribonuclease protein-like	*Saccoglossus kowalevskii*
Unigene0052995	Hypothetical transcript	*Hymenolepis microstoma*
Unigene0020574	Endonuclease-reverse transcriptase	*Lasius niger*
Unigene0062719	Reverse transcriptase	*Lasius niger*
Unigene0067624	Transposon ty3-i gag-pol polyprotein	*Lasius niger*
Unigene0068232	RNA-directed dna polymerase from mobile element jockey-like protein, partial	*Lasius niger*
Unigene0052994	rRNA promoter binding protein	*Echinococcus granulosus*
Unigene0077568	rRNA promoter binding protein	*Echinococcus granulosus*
Unigene0052994	rRNA promoter binding protein	*Echinococcus granulosus*
Unigene0055574	Transposon Ty3-I Gag-Pol polyprotein, partial	*Exaiptasia pallida*
Unigene0075830	Retrovirus-related Pol polyprotein from transposon 17.6	*Exaiptasia pallida*
Unigene0010682	Pro-Pol polyprotein	*Exaiptasia pallida*
Unigene0028718	Integrator complex subunit 4	*Exaiptasia pallida*
Unigene0029489	Retrovirus-related Pol polyprotein from transposon opus	*Exaiptasia pallida*
Unigene0075682	Hypothetical protein AC249_AIPGENE24709	*Exaiptasia pallida*
Unigene0045545	Integrase/recombinase xerD-like, partial	*Exaiptasia pallida*
Unigene0022024	Fibrillin-1	*Exaiptasia pallida*
Unigene0013991	Neurotrypsin	*Exaiptasia pallida*
Unigene0033842	Retrovirus-related Pol polyprotein from type-1 retrotransposable element R2	*Exaiptasia pallida*
Unigene0016956	Retrovirus-related Pol polyprotein from transposon 17.6	*Exaiptasia pallida*
Unigene0022248	Transposon TX1 uncharacterized 149 kDa protein	*Exaiptasia pallida*

Unigene0074252	RNA-directed DNA polymerase from mobile element jockey-like	*Strongylocentrotus purpuratus*
Unigene0012856	RNA-directed DNA polymerase from mobile element jockey-like	*Strongylocentrotus purpuratus*
Unigene0075941	Uncharacterized protein LOC100891119	*Strongylocentrotus purpuratus*
Unigene0064171	Cysteine sulfinic acid decarboxylase	*Litopenaeus vannamei*
Unigene0057678	Alkaline phosphatase	*Litopenaeus vannamei*
Unigene0065508	ZAN protein precursor	*Ciona intestinalis*
Unigene0026012	Fibronectin-like, partial	*Ciona intestinalis*
Unigene0064326	Disrupted in renal carcinoma protein 2	*Daphnia magna*
Unigene0073362	Chorion peroxidase precursor	*Daphnia magna*
Unigene0063941	Daphnid bacterial-ribosomal-RNA-like, possible HGT	*Daphnia magna*
Unigene0061920	Hypothetical protein, partial	*Daphnia magna*
Unigene0024515	Cathepsin L, partial	*Daphnia magna*
Unigene0005355	Enzymatic polyprotein	*Daphnia magna*
Unigene0064045	Peroxisome biogenesis factor 10	*Daphnia magna*
Unigene0041112	Hypothetical protein NEMVEDRAFT_v1g142724	*Nematostella vectensis*
Unigene0036248	Predicted protein	*Nematostella vectensis*
Unigene0004359	14-3-3 protein, partial	*Penaeus monodon*
Unigene0042568	Thromboxane A synthase	*Penaeus monodon*
Unigene0002542	StAR-related lipid transfer protein 3	*Penaeus monodon*
Unigene0063752	Vitellogenin	*Pandalus hypsinotus*
Unigene0028810	Apolipoprotein D	*Zootermopsis nevadensis*
Unigene0020378	G-protein coupled receptor Mth2	*Zootermopsis nevadensis*
Unigene0065672	Proton-coupled amino acid transporter 4	*Zootermopsis nevadensis*
Unigene0075897	Chorion peroxidase	*Microplitis demolitor*
Unigene0032105	Pprobable glycosidase CRH1, partial	*Hydra vulgaris*
Unigene0053285	RNA-directed DNA polymerase from mobile element jockey-like	*Hydra vulgaris*
Unigene0050615	Uncharacterized protein C2orf16-like	*Hydra vulgaris*
Unigene0074604	Dipeptidase 1-like, partial	*Cephus cinctus*
Unigene0048607	Protein PBMUCL2	*Melipona quadrifasciata*
Unigene0052257	Cdc42 like protein	*Melipona quadrifasciata*
Unigene0074075	Hypothetical protein HELRODRAFT_158911	*Helobdella robusta*
Unigene0039229	Hypothetical protein T02_16311	*Trichinella nativa*
Unigene0027377	PiggyBac transposable element-derived protein 4-like	*Amyelois transitella*
Unigene0065511	Uncharacterized protein LOC100568563	*Acyrthosiphon pisum*
Unigene0041961	RNA-directed DNA polymerase from mobile element jockey-like	*Acyrthosiphon pisum*

Unigene0030242	SCAN domain-containing protein 3-like	*Acyrthosiphon pisum*
Unigene0069719	Reverse transcriptase	*Daphnia pulex*
Unigene0041647	Reverse transcriptase, partial	*Nuttalliella namaqua*
Unigene0050597	Hypothetical protein M514_07931	*Trichuris suis*
Unigene0055353	Ferlin [Plasmodium cynomolgi strain B]	*Plasmodium cynomolgi*
Unigene0050616	Mucin-5AC-like	*Diaphorina citri*
Unigene0050619	Mucin-5AC-like	*Diaphorina citri*
Unigene0025349	RNA-directed DNA polymerase from mobile element jockey-like	*Diaphorina citri*
Unigene0005091	Craniofacial development protein 2-like, partial	*Diaphorina citri*
Unigene0007503	Peroxidase-like	*Diaphorina citri*
Unigene0045903	Endonuclease-reverse transcriptase	*Danaus plexippus*
Unigene0055798	Pro-resilin-like	*Diuraphis noxia*
Unigene0029960	SCAN domain-containing protein 3-like	*Diuraphis noxia*
Unigene0029918	Tubulin	*Tetrahymena thermophila SB210*
Unigene0073421	Tubulin/FtsZ family, GTPase domain protein	*Tetrahymena thermophila SB210*
Unigene0076277	Hormone receptor 4-like	*Aplysia californica*
Unigene0050617	Hypothetical protein LOTGIDRAFT_72903, partial	*Lottia gigantea*
Unigene0030615	Hypothetical protein LOTGIDRAFT_172470	*Lottia gigantea*
Unigene0021111	Tigger transposable element-derived protein 1-like	*Octopus bimaculoides*
Unigene0029175	Serine protease	*Scylla paramamosain*
Unigene0066327	RNA-directed DNA polymerase (reverse transcriptase) domain containing protein	*Haemonchus contortus*
Unigene0048616	Kazal-type serine proteinase inhibitor 4, partial	*Procambarus clarkii*
Unigene0013707	Methyltransferase-like protein, partial	*Latrodectus hesperus*
Unigene0034329	Estrogen sulfotransferase	*Scylla olivacea*
Unigene0036440	Estrogen sulfotransferase	*Scylla olivacea*
Unigene0009430	Estrogen sulfotransferase	*Scylla olivacea*
Unigene0027998	General transcription factor IIH subunit 4	*Wasmannia auropunctata*
Unigene0010081	Zinc metalloproteinase nas-4-like	*Wasmannia auropunctata*
Unigene0062188	C-reactive protein 1.1-like	*Limulus polyphemus*
Unigene0055390	Cornifin-B-like, partial	*Limulus polyphemus*
Unigene0041371	Mite allergen Lep d 7-like, partial	*Limulus polyphemus*
Unigene0015713	LOW QUALITY PROTEIN: arylsulfatase B-like	*Limulus polyphemus*

Unigene0013082	Collagen alpha-1(I) chain-like	*Limulus polyphemus*
Unigene0068101	Lysosomal aspartic protease	*Cerapachys biroi*
Unigene0072629	PiggyBac transposable element-derived protein 4-like	*Papilio xuthus*
Unigene0004694	Probable H/ACA ribonucleoprotein complex subunit 1 precursor	*Papilio xuthus*
Unigene0009236	Reverse transcriptase, partial	*Anopheles arabiensis*
Unigene0041646	Golgin subfamily A member 6-like protein 22	*Acropora digitifera*
Unigene0028734	TNF receptor-associated factor 5-like isoform X2	*Acropora digitifera*
Unigene0003085	RNA-directed DNA polymerase from mobile element jockey-like	*Acropora digitifera*
Unigene0018800	Astacin-like metalloprotease toxin 5	*Acropora digitifera*
Unigene0026600	Zinc finger protein 36, C3H1 type-like 1, partial	*Stegodyphus mimosarum*
Unigene0020381	G-protein coupled receptor Mth2-like isoform X3	*Monomorium pharaonis*
Unigene0028497	Voltage-dependent calcium channel gamma-5 subunit	*Tribolium castaneum*
Unigene0006138	Microtubule-actin cross-linking factor 1 isoform X24	*Dinoponera quadriceps*
Unigene0068154	Tubulin alpha-1 chain, partial	*Trichinella murrelli*
Unigene0056892	Fibrillin-3-like	*Lingula anatina*
Unigene0066955	Nose resistant to fluoxetine protein 6-like isoform X2	*Lingula anatina*
Unigene0050124	Multiple epidermal growth factor-like domains protein 8	*Biomphalaria glabrata*
Unigene0006133	Transposase, ISXO2-like domain-containing protein	*Strongyloides ratti*
Unigene0036754	Sperm gelatinase-like protease	*Macrobrachium rosenbergii*
Unigene0022901	Male reproductive-related protein A	*Macrobrachium rosenbergii*
Unigene0060515	Male reproductive-related protein A	*Macrobrachium rosenbergii*
Unigene0010280	Crustin	*Macrobrachium rosenbergii*
Unigene0022902	Male reproductive-related protein A	*Macrobrachium rosenbergii*
Unigene0026567	Uncharacterized protein LOC107217456	*Neodiprion lecontei*
Unigene0011508	Retrovirus-related Pol polyprotein from type-1 retrotransposable element R2, partial	*Toxocara canis*

Unigene0065550	Vitellogenin 2	*Pandalopsis japonica*
Unigene0058771	Chitinase	*Pandalopsis japonica*
Unigene0052411	Vitellogenin 2	*Pandalopsis japonica*
Unigene0023257	Gag-Pol polyprotein	*Trachymyrmex cornetzi*
Unigene0023631	Matrix metalloproteinase 9, partial	*Paracyclopina nana*
Unigene0023628	Kielin/chordin-like protein isoform X2	*Pogonomyrmex barbatus*
Unigene0001122	Kazal-type protease inhibitor	*Macrobrachium nipponense*
Unigene0000359	Kazal-type protease inhibitor	*Macrobrachium nipponense*
Unigene0003459	Uncharacterized protein LOC105397399	*Plutella xylostella*
Unigene0004520	Aminopeptidase N	*Trichogramma pretiosum*
Unigene0008214	Hemocyanin	*Palaemon carinicauda*
Unigene0036597	Hemocyanin	*Palaemon carinicauda*
Unigene0016779	Vitellogenin	*Palaemon carinicauda*
Unigene0022977	Facilitated trehalose transporter Tret1 isoform X1	*Athalia rosae*
Unigene0016233	Hypothetical protein ALC62_05715	*Cyphomyrmex costatus*
Unigene0060722	Facilitated trehalose transporter Tret1-like	*Cimex lectularius*
Unigene0013764	Tubulin alpha-1 chain-like	*Halyomorpha halys*
Unigene0029127	TPPP family protein CG45057 isoform X1	*Drosophila ficusphila*
Unigene0015995	Uncharacterized protein LOC105336494	*Crassostrea gigas*
Unigene0033404	Cathepsin B	*Pandalus borealis*
