# Supplementary material for: Transcriptome Analysis Provides Insights into the Mechanism of Astaxanthin Enrichment in a Mutant of the Ridgetail White Prawn Exopalaemon carinicauda
Source: Genes (Basel). 2021 Apr 21;12(5):618. doi: 10.3390/genes12050618 (PMC8143343; doi:10.3390/genes12050618)
Supplement: Supplementary file 1 [file genes-12-00618-s001.zip › Table S2.pdf]

Table S2. Information of DEGs with lower expression level in EcR prawns.

GeneID	Description	Species
Unigene0041530	Endonuclease-reverse transcriptase	*Lasius niger*
Unigene0055566	Transposable element tc3 transposase	*Lasius niger*
Unigene0004363	Single insulin-like growth factor-binding domain protein-2	*Cupiennius salei*
Unigene0061823	Cuticular protein analogous to peritrophins 1-F, partial	*Daphnia magna*
Unigene0044620	Apolipoprotein D	*Daphnia magna*
Unigene0047842	Lathosterol oxidase	*Daphnia magna*
Unigene0001081	Hypothetical protein, partial	*Daphnia magna*
Unigene0035595	Cuticle protein, partial	*Daphnia magna*
Unigene0063944	Daphnid bacterial-ribosomal-RNA-like, possible HGT	*Daphnia magna*
Unigene0026083	Uncharacterized protein APZ42_034555	*Daphnia magna*
Unigene0056165	Pro-resilin, partial	*Daphnia magna*
Unigene0036561	Apolipoprotein D	*Daphnia magna*
Unigene0066106	Heparan-alpha-glucosaminide N-acetyltransferase	*Daphnia magna*
Unigene0052919	DNA ligase	*Daphnia magna*
Unigene0063938	Daphnid bacterial-ribosomal-RNA-like, possible HGT, partial	*Daphnia magna*
Unigene0023217	AGAP006931-PA	*Anopheles gambiae*
Unigene0041205	AGAP002074-PA	*Anopheles gambiae*
Unigene0001866	Gamma-interferon induced thiol reductase GILT3	*Scylla paramamosain*
Unigene0057102	Vitelline membrane outer layer 1-like protein	*Scylla paramamosain*
Unigene0004252	Single VWC domain protein 1	*Penaeus monodon*
Unigene0038524	Single VWC domain protein 1	*Penaeus monodon*
Unigene0023760	Tetraspanins-like protein	*Penaeus monodon*
Unigene0008877	Tyrosine-protein phosphatase 10D, partial	*Zootermopsis nevadensis*
Unigene0016520	Phosphoinositide 3-kinase adapter protein 1, partial	*Zootermopsis nevadensis*
Unigene0006879	Hypothetical protein L798_05011, partial	*Zootermopsis nevadensis*
Unigene0022872	N-acetyl-beta-D-glucosaminidase	*Macrobrachium nipponense*
Unigene0027904	Hemocyanin	*Macrobrachium nipponense*
Unigene0017828	Delta-6 desaturase	*Macrobrachium nipponense*
Unigene0008712	Pupal cuticle protein	*Cephus cinctus*
Unigene0046800	Pupal cuticle protein	*Cephus cinctus*
Unigene0054046	L-lactate dehydrogenase-like	*Cephus cinctus*
Unigene0053211	Zinc finger RNA-binding protein, partial	*Stegodyphus mimosarum*

Unigene0068209	PREDICTED: zinc finger CCCH Domain-containing protein 11A-like	*Saccoglossus kowalevskii*
Unigene0044808	LOW QUALITY PROTEIN: probable E3 ubiquitin-protein	*Saccoglossus kowalevskii*
Unigene0005526	GL12602	*Drosophila persimilis*
Unigene0013440	Cubilin-like	*Bombyx mori*
Unigene0019752	Cuticular protein RR-2 motif 78 precursor	*Bombyx mori*
Unigene0002837	Carboxypeptidase inhibitor precursor	*Bombyx mori*
Unigene0033209	Gastrolith protein 18.2	*Cherax quadricarinatus*
Unigene0029526	Gastrolith protein 30	*Cherax quadricarinatus*
Unigene0050714	Post-molt protein 1	*Cherax quadricarinatus*
Unigene0032773	Cuticle protein 1	*Cherax quadricarinatus*
Unigene0053211	Zinc finger RNA-binding protein, partial	*Stegodyphus mimosarum*
Unigene0065726	Sentrin-specific protease 1	*Trachymyrmex septentrionalis*
Unigene0040009	Hypothetical protein BRAFLDRAFT_63711	*Branchiostoma floridae*
Unigene0059308	Hypothetical protein BRAFLDRAFT_77273	*Branchiostoma floridae*
Unigene0059309	Hypothetical protein BRAFLDRAFT_106667	*Branchiostoma floridae*
Unigene0026889	Hypothetical protein BRAFLDRAFT_63711	*Branchiostoma floridae*
Unigene0030980	Hypothetical protein BRAFLDRAFT_221813	*Branchiostoma floridae*
Unigene0067838	Hypothetical protein HELRODRAFT_164914	*Helobdella robusta*
Unigene0049424	Crustapain	*Pandalus borealis*
Unigene0034568	C-type lectin	*Procambarus clarkii*
Unigene0016894	C-type lectin	*Procambarus clarkii*
Unigene0022558	Strongly chitin-binding protein-1	*Procambarus clarkii*
Unigene0037661	Calcification-associated peptide-2	*Procambarus clarkii*
Unigene0039421	Calcification-associated peptide-2	*Procambarus clarkii*
Unigene0024487	Reverse transcriptase	*Daphnia pulex*
Unigene0064526	Cuticle protein 6	*Blaberus craniifer*
Unigene0023218	Cuticle protein 6	*Blaberus craniifer*
Unigene0017067	Gamma-butyrobetaine dioxygenase-like isoform X2	*Lingula anatina*
Unigene0052337	Interferon alpha-inducible protein 27-like protein 2B	*Lingula anatina*
Unigene0023090	Interferon alpha-inducible protein 27-like protein 2A isoform X1	*Lingula anatina*
Unigene0040810	Uncharacterized 51.9 kDa protein in	*Lingula anatina*

	rps4-rps11 intergenic region-like	
Unigene0077067	Prolyl tripeptidyl peptidase-like	*Lingula anatina*
Unigene0000485	Uncharacterized protein LOC107166243	*Diuraphis noxia*
Unigene0001792	Somatostatin receptor type 2-like	*Diuraphis noxia*
Unigene0044112	Monocarboxylate transporter 14-like	*Halyomorpha halys*
Unigene0059050	Esterase E4-like	*Litopenaeus vannamei*
Unigene0019159	Glucose transporter 2	*Litopenaeus vannamei*
Unigene0005817	Single VWC domain protein 1	*Litopenaeus vannamei*
Unigene0018100	Cathepsin L2, partial	*Litopenaeus vannamei*
Unigene0017001	Ecdysteroid regulated-like protein	*Litopenaeus vannamei*
Unigene0058008	Single VWC domain protein 1	*Litopenaeus vannamei*
Unigene0059703	Androgenic gland-specific protein	*Macrobrachium rosenbergii*
Unigene0077051	VLIG2	*Macrobrachium rosenbergii*
Unigene0042919	Putative articulin p60, partial	*Rimicaris exoculata*
Unigene0040043	Histone-lysine N-methyltransferase SETMAR-like	*Limulus polyphemus*
Unigene0013204	Ryncolin-2-like	*Amphimedon queenslandica*
Unigene0057925	RNA exonuclease 1 like protein	*Melipona quadrifasciata*
Unigene0033331	RNA exonuclease 1 like protein	*Melipona quadrifasciata*
Unigene0013490	CUB domain-containing protein	*Oryctes borbonicus*
Unigene0046960	C-type lectin-like domain-containing protein PtLP	*Portunus trituberculatus*
Unigene0056293	C-type lectin-like domain-containing protein PtLP	*Portunus trituberculatus*
Unigene0054358	C-type lectin 2	*Marsupenaeus japonicus*
Unigene0029470	DD5	*Marsupenaeus japonicus*
Unigene0019787	DD5	*Marsupenaeus japonicus*
Unigene0015318	Lectin C, partial	*Marsupenaeus japonicus*
Unigene0029471	DD5	*Marsupenaeus japonicus*
Unigene0044640	DD9A, partial	*Marsupenaeus japonicus*
Unigene0001656	Obstructor B	*Locusta migratoria*
Unigene0040077	CUB-serine protease	*Panulirus argus*
Unigene0052190	Olfactory ionotropic receptor IR4	*Panulirus argus*
Unigene0003529	Cuticular protein	*Tenebrio molitor*
Unigene0006726	C-type lectin	*Ruditapes philippinarum*
Unigene0021532	Serine proteinase stubble	*Bactrocera dorsalis*
Unigene0031612	PVenom protein 302-like	*Parasteatoda tepidariorum*
Unigene0047109	Zinc finger protein OZF-like	*Parasteatoda tepidariorum*
Unigene0060941	Anaphase-promoting complex subunit 10-like	*Parasteatoda tepidariorum*
Unigene0066088	Tripartite motif-containing 13-like, partial	*Strongylocentrotus purpuratus*
Unigene0054180	Crustacyanin subunit A	*Fenneropenaeus merguiensis*
Unigene0055661	Tenascin-R	*Crassostrea gigas*

Unigene0023066	Hypothetical protein CGI_10021602	*Crassostrea gigas*
Unigene0018807	RNA-directed DNA polymerase (reverse transcriptase) domain containing protein	*Haemonchus contortus*
Unigene0004592	Uncharacterized protein LOC107334107	*Acropora digitifera*
Unigene0031789	Protein UBASH3A homolog isoform X2	*Polistes canadensis*
Unigene0040042	Uncharacterized protein LOC106815379	*Priapulus caudatus*
Unigene0026156	Circumsporozoite protein-like	*Priapulus caudatus*
Unigene0018213	Hypothetical protein ALC62_05268	*Cyphomyrmex costatus*
Unigene0027281	Keratin-associated protein 4-4-like, partial	*Biomphalaria glabrata*
Unigene0014937	Methionine adenosyltransferase, partial	*Ophiopholis sp. KP-2011*
Unigene0076836	FLYWCH-type zinc finger-containing protein 1	*Toxocara canis*
Unigene0043447	Cuticular protein 34	*Eriocheir sinensis*
Unigene0017103	Cuticular protein 34	*Eriocheir sinensis*
Unigene0037069	Cuticular protein 34	*Eriocheir sinensis*
Unigene0035142	Cuticular protein 34	*Eriocheir sinensis*
Unigene0073146	Cuticular protein 34	*Eriocheir sinensis*
Unigene0004362	TPA_inf: hypothetical secreted protein 323	*Amblyomma variegatum*
Unigene0064815	Neprilysin-2-like	*Cerapachys biroi*
Unigene0070933	Annexin B11-like Protein	*Tribolium castaneum*
Unigene0021752	DnaK protein	*Trichomonas vaginalis G3*
Unigene0066767	Hypothetical protein M514_21119, partial	*Trichuris suis*
Unigene0026784	Uncharacterized protein LOC106872177	*Octopus bimaculoides*
Unigene0025406	Cuticle protein CP1876	*Cancer pagurus*
Unigene0066169	RNA-directed DNA polymerase from mobile element jockey-like	*Hydra vulgaris*
Unigene0028426	Hypothetical protein LOTGIDRAFT_153639	*Lottia gigantea*
Unigene0028670	Endonuclease-reverse transcriptase	*Danaus plexippus*
Unigene0062264	Hypothetical protein CAPTEDRAFT_111329, partial	*Capitella teleta*
Unigene0045952	Perlucin-like	*Aplysia californica*
Unigene0016948	cuticle protein BD2	*Portunus pelagicus*
Unigene0074159	Chitinase	*Operophtera brumata*
Unigene0053792	Hypothetical protein EPH_0032430	*Eimeria praecox*
Unigene0075485	Uncharacterized protein LOC105675530	*Linepithema humile*
Unigene0074903	Ribosomal protein L40	*Hymenolepis microstoma*
Unigene0000910	Beta-crystallin A1	*Lepeophtheirus salmonis*
Unigene0010114	Calcified cuticle protein CP19.0 isoform A	*Callinectes sapidus*
Unigene0042544	Calcified cuticle protein CP19.0 isoform	*Callinectes sapidus*

	B	
Unigene0072453	Calcified cuticle protein CP19.0 isoform B	*Callinectes sapidus*
Unigene0019577	Calcified cuticle protein CP14.1	*Callinectes sapidus*
Unigene0038920	Cuticle proprotein, partial	*Palaemon varians*
Unigene0040809	Nesprin-1 isoform X2	*Drosophila miranda*
