# Supplementary material for: Transcriptome Analysis Provides Insights into the Mechanism of Astaxanthin Enrichment in a Mutant of the Ridgetail White Prawn Exopalaemon carinicauda
Source: Genes (Basel). 2021 Apr 21;12(5):618. doi: 10.3390/genes12050618 (PMC8143343; doi:10.3390/genes12050618)
Supplement: Supplementary file 1 [file genes-12-00618-s001.zip › Table S3.pdf]

Table S3. Information of lipoproteins used in phylogenetic analysis.

Name	Species	Accession Number
apoCr	*Penaeus semisulcatus*	AY051318
	Marsupenaeus japonicus	AB033719
	Metapenaeus ensis	AY103478
	Metapenaeus ensis	AY530205
	Litopenaeus vannamei	AY321153
	Fenneropenaeus merguiensis	AY499620
	Penaeus monodon	DQ288843
	Pandalus hypsinotus	AB117524
	Cherax quadricarinatus	AF306784
	Macrobrachium rosenbergii	AB056458
	Charybdis feriatus	AY724676
	Portunus trituberculatus	AAX94762
apoLp-II/I	*Locusta migratoria*	Q9U943
	Manduca sexta	Q25490
	Drosophila melanogaster	Q9V496
	Apis mellifera	XP_392490
	Anopheles gambiae	XP_321226
	Samia cynthia ricini	BAB32641
apoB	*Danio rerio*	XP_694827
	Gallus gallus	XP_419979
	Homo sapiens	X04714
CP	*Pacifastacus leniusculus*	AAD16454
	Penaeus monodon	AAF19002
MTP	*Drosophila melanogaster*	NP_610075
	Anopheles gambiae	XP_319421
	Caenorhabditis elegans	AAR27937
	Strongylocentrotus purpuratusthe	XP_788526
	Danio rerio	Q8AXV7
	Bos taurus	X78567
	Homo sapiens	P55157
Vtg	*Crassostrea gigas*	BAC22716
	Mizuhopecten yessoensis	BAB63260
	Caenorhabditis elegans	P55155
	Caenorhabditis elegans	P06125
	Caenorhabditis elegans	P18948
	Oscheius sp.	T18561
	Ichthyomyzon unicuspis	Q91062
	Acipenser transmontanus	Q90243
	Fundulus heteroclitus	U07055
	Oncorhynchus mykiss	Q92093
	Xenopus laevis	P18709
	Gallus gallus	P87498
